# Primary, Secondary, and Tertiary Effects of Carbohydrate Ingestion During Exercise

**DOI:** 10.1007/s40279-020-01343-3

**Published:** 2020-09-16

**Authors:** Ian Rollo, Javier T. Gonzalez, Cas J. Fuchs, Luc J. C. van Loon, Clyde Williams

**Affiliations:** 1 Gatorade Sports Science Institute, PepsiCo Life Sciences, Global R&D, Leicestershire, UK; 2 School of Sports Exercise and Health Sciences, Loughborough University, Loughborough, UK; 3 Department for Health, University of Bath, Bath, UK; 4 Department of Human Biology, NUTRIM School of Nutrition and Translational Research in Metabolism, Maastricht University Medical Centre+, Maastricht, The Netherlands

## Abstract

The purpose of this current opinion paper is to describe the journey of ingested carbohydrate from ‘mouth to mitochondria’ culminating in energy production in skeletal muscles during exercise. This journey is conveniently described as primary, secondary, and tertiary events. The primary stage is detection of ingested carbohydrate by receptors in the oral cavity and on the tongue that activate reward and other centers in the brain leading to insulin secretion. After digestion, the secondary stage is the transport of monosaccharides from the small intestine into the systemic circulation. The passage of these monosaccharides is facilitated by the presence of various transport proteins. The intestinal mucosa has carbohydrate sensors that stimulate the release of two ‘incretin’ hormones (GIP and GLP-1) whose actions range from the secretion of insulin to appetite regulation. Most of the ingested carbohydrate is taken up by the liver resulting in a transient inhibition of hepatic glucose release in a dose-dependent manner. Nonetheless, the subsequent increased hepatic glucose (and lactate) output can increase exogenous carbohydrate oxidation rates by 40–50%. The recognition and successful distribution of carbohydrate to the brain and skeletal muscles to maintain carbohydrate oxidation as well as prevent hypoglycaemia underpins the mechanisms to improve exercise performance.

## Key Points

**Table:** 

Receptors in the oral cavity detect ingested carbohydrate and activate reward and other centers in the brain that can improve sports performance.
Exogenous carbohydrate availability and subsequent carbohydrate oxidation rates can be increased by co-ingesting fructose with glucose (polymers).
Ingested carbohydrate provides fuel for the brain as well as working skeletal muscle tissue. Its distribution via the liver is orchestrated, so that hypoglycaemia and fatigue are delayed.

## Introduction

Since the recognition that fatigue during prolonged exercise is accompanied by the depletion of muscle glycogen stores [1], nutritional studies have been directed at increasing pre-exercise carbohydrate (CHO) stores as well as providing additional CHO during exercise.

Strategies to increase pre-exercise muscle glycogen stores include increasing daily dietary CHO intake [1–4] and ingesting CHO-rich meals prior to exercise. The consumption of easy-to-digest CHO-rich meals 3 h before exercise increases liver and muscle glycogen concentrations by 11–15% [5, 6]. Commencing exercise with elevated liver and muscle glycogen contents can improve endurance capacity, as has been consistently shown with running and cycling exercise [6–8]. When a pre-exercise CHO-rich meal is combined with the ingestion of CHO during exercise, then the improvements in endurance capacity during prolonged cycling [9] and during running [10] tasks are greater than when either of these CHO interventions is adopted separately. Endurance time-trial performance (~ 1 h > 70% $\dot{V}$O_2_max) has also been reported to benefit from CHO ingestion during running and cycling exercise [11, 12]. However, the magnitude of benefit likely depends on the pre-exercise endogenous CHO storage levels of an individual [13].

The ingestion of CHO has been shown to improve endurance capacity by maintaining euglycemia late in exercise and, under certain circumstances, delaying the depletion of muscle glycogen stores [14, 15]. Other mechanisms by which CHO may influence endurance performance may be “centrally” mediated [16]. The central and peripheral effects of CHO ingestion are not mutually exclusive, as CHO must pass through the gastrointestinal (GI) tract before entering the peripheral circulation. Thus, the mechanisms by which CHO influences exercise performance go beyond simply providing enough substrate for energy metabolism.

The purpose of this current opinion paper is to classify the effect of CHO ingestion into three major stages; primary, secondary, and tertiary. This paper will address these stages in sequence discussing how the delivery of (or delivered) substrate for energy metabolism during exercise impacts on performance.

## Primary

Endogenous CHO stores within the liver and skeletal muscles are relatively limited. Therefore, to maintain the supply of energy for muscle and brain function, CHO stores are needed to be replenished frequently. This is achieved through our habitual diet. In line, most sports nutrition interventions include the ingestion of CHO-rich foods or food supplements. Therefore, the effectiveness of nutritional interventions is dependent upon the absorption of CHO by the GI tract.

The digestion of CHO begins in the mouth. Food is broken down mechanically by mastication and mixed with saliva. Salivary amylase begins the breakdown of ingested starch into small oligosaccharides. In the mouth, the tongue begins with the analysis of food, determining whether it is nutritive (i.e., contains available carbohydrates) and should be ingested or whether it is potentially harmful and should be expectorated [17]. Sweet stimuli (glucose, sucrose, fructose, and artificial sweeteners) are detected by taste receptor cells (G-coupled receptor proteins; T1R2 and T1R3) on the tongue [18]. These receptor cells release a neurotransmitter (α-gustducin) that is detected by primary afferent nerve fiber terminals, sending information to the brainstem. The central processing of sweet taste activates feeding circuits as well as brain reward systems that promote sweet appetite [18]. The palatability of solutions is an important consideration when investigating carbohydrate–electrolyte (CHO-E) beverages. Several studies have reported that flavoured or sweetened beverages can substantially increase the voluntary intake of fluid both during exercise and throughout subsequent recovery [19, 20].

The central response to ingesting CHO has been investigated using functional magnetic resonance imaging (*f*MRI). In one study, participants ingested either 300 mL of water (control), a glucose solution, an aspartame (sweet taste) solution, or a maltodextrin (non-sweet CHO) solution. Both sweet taste and energy content appeared to produce a hypothalamic response [21]. The hypothalamic response was reported to be dose dependent on CHO, specifically, in relation to changes in circulating insulin concentrations [22].

Both glucose (sweet) and glucose polymers (non-sweet) in the mouth activate regions in the brain associated with reward, such as the insula/frontal operculum, orbitofrontal cortex, and striatum. These findings suggest that there may be a class of, so far unidentified, oral receptors that respond to CHO independently of sweetness [23]. Regions of the brain associated with reward are also believed to mediate behavioral responses to rewarding stimuli, such as taste [24].

Receptors on the tongue also extract information about the texture and temperature of food. This processing prepares the GI system for compounds in the mouth by causing the organism to salivate, masticate, swallow, or expel, as well as to release a cascade of post-prandial hormones such as insulin and other peptides [17]. In humans, simply tasting food stimulates the release of insulin from the pancreas, known as the cephalic insulin release (CPIR). Under fasting conditions, both nutritive (sucrose) and non-nutritive sweetener (saccharin) solutions have been shown to induce CPIR, when mouth-rinsed for 45 s and expectorated without ingestion [25]. However, the magnitude of change in insulin secretion from the pancreas following the CPIR is negligible (1–2 mU/L) compared to the ~ 80-fold increase in response to elevated blood glucose concentrations. Although it is not known if the CPIR persists during exercise, it is unlikely to impact on carbohydrate metabolism or performance.

Rinsing a CHO solution (6–10%) for 5–10 s in the mouth during exercise has been reported to increase both endurance [26] and strength performance [27]. However, it should be noted that this is not confirmed by all the studies [28, 29]. The apparent discrepancies may be attributed to differences in pre-exercise CHO status of participants and the inability of the protocols applied to detect small differences in exercise performance. Nevertheless, the detection of CHO in the mouth exerts a primary effect during exercise by modulating feelings of pleasure/displeasure, lowering the perception of effort during an exercise task and/or facilitating the recruitment of additional motor units in working skeletal muscles [30–33].

## Secondary

The secondary effect(s) of CHO ingestion on exercise performance occur between the absorption of CHO across the intestine to the availability of the monosaccharides in the systemic circulation. After leaving the stomach, CHO enters the small intestine where disaccharides, oligosaccharides, and polysaccharides must first be hydrolysed to their constituent monosaccharides before subsequent absorption and utilization [34]. Ingesting CHO in solution results in faster gastric emptying rates when compared with the ingestion of solid foods [35]. Dilute CHO solutions (e.g., up to 6% or 60 g carbohydrate/L) are emptied from the stomach at a similar rate as an equal volume of water [36]. By contrast, increasing the concentration of CHO in a solution to ≥ 8% and modifying the CHO type (glucose versus equivalent amounts of galactose or fructose [37]) can significantly impair gastric emptying. It is thought that mechanisms leading to delayed gastric emptying act via the stimulation of osmoreceptors in the duodenum [38]. Gastric emptying rates of 4–8% carbohydrate solutions are not thought to be rate limiting to intestinal CHO absorption and subsequent oxidation during exercise [39]. Therefore, differences in performance seen by modulating the type and amount of CHO ingested during exercise are likely to act via mechanisms other than gastric emptying.

Carbohydrates are taken up in the GI tract by transporter proteins located on the brush borders of the intestinal membrane. Glucose and galactose are primarily absorbed via active transport of Na^+^ by the sodium-dependent luminal transport protein (SGLT1; *SLC5A1*) [40]. In contrast, fructose is primarily absorbed by the protein carrier, GLUT-5 (*SLC2A5*), which is not Na^+^ dependent [34, 41]. Whilst other transporters have been suggested to play a role in glucose and/or fructose absorption, such as GLUT2, GLUT8, and GLUT12, there is little evidence that these transport proteins play quantitatively important roles in carbohydrate absorption across the apical membrane in humans [42, 43]. It appears that the SGLT1 transport pathway is saturated when ingesting more than 1.2 g glucose (polymers) per min, thereby restricting maximum exogenous glucose oxidation rates to 1.0–1.2 g glucose per min. However, the combined ingestion of fructose with glucose allows the use of both intestinal transport pathways to increase the total exogenous CHO oxidation rates during exercise to up to ~ 1.7 g exogenous carbohydrate per min [44, 45]. It is important to emphasise that CHO ingestion rates required to elicit peak exogenous oxidation rates are very high and relevant only to prolonged excise (2.5–3 h) performed by highly trained athletes [46] (Fig. 3). Enhancing intestinal CHO absorption rates increases exogenous CHO availability for key tissues such as the liver and muscle, and lowers the gastrointestinal distress associated with the ingestion of large quantities of CHO [47, 48].

Endurance-type exercise can produce splanchnic hypoperfusion, which results in a rapid increase in the concentration of plasma intestinal fatty acid-binding protein (I-FABP) [49], a marker of intestinal epithelial cell turnover and integrity. The functional effects of exercise-induced increases in plasma I-FABP are not entirely clear, although there may be implications for gut barrier dysfunction and/or post-exercise nutrient processing [49]. The ingestion of a CHO before [50] or during exercise [51] reduces splanchnic hypoperfusion and prevents the increase in plasma I-FABP concentrations during exercise. Therefore, CHO availability in the intestine may play a role in regulating intestinal epithelial cell integrity.

The intestinal mucosa is also involved in CHO sensing. The potential for the gut to sense glucose was established when it was observed that glucose ingestion produces a greater insulin response when compared to intravenous glucose infusion, matched for circulating plasma glucose concentrations [52]. This response has been termed the incretin effect and is primarily a result of the secretion of two incretin hormones: glucose-dependent insulinotropic polypeptide (GIP) and glucagon-like peptide-1 (GLP-1). The absorption of CHO via the SGLT1 transporter is thought to be a key intestinal glucose-sensing mechanism. Substrates for SGLT1 such as glucose, galactose, and glucose analogues stimulate GIP secretion, and these responses can be abolished by the addition of phloridizin, a competitive inhibitor of SGLT1 [53]. These incretin hormones display a variety of physiological effects, from insulin secretion to appetite regulation, yet their roles in exercise metabolism are currently unclear. In addition to hormonal signaling, intestinal sensing of glucose may also signal via vagal afferent pathways [54], yet the implications of such sensing for exercise metabolism remains to be elucidated. Following intestinal absorption, ingested CHO are transported to the liver via the portal vein, where glucoreceptors are present. These glucoreceptors are innervated by vagal afferent fibers and are important in the sympathoadrenal response to hypoglycaemia [55]. Information from peripheral glucose sensors along the GI tract converges in the nucleus solitarius of the caudal medulla of the brain. The information provided is used by the medulla to generate appropriate oropharyngeal and autonomic motor responses, and can be relayed to the hypothalamus and taste/visceral cortex via the lateral parabranchial nucleus in the pons [18].

**Fig. 1:**
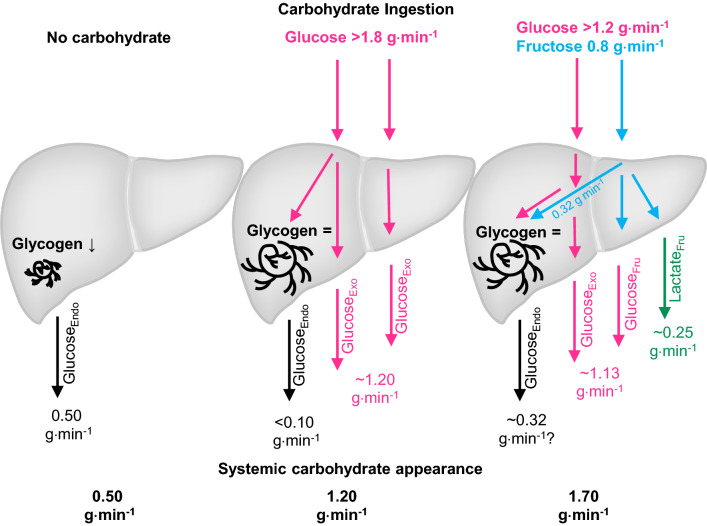
Influence of carbohydrate ingestion on carbohydrate appearance in the systemic circulation. In the absence of carbohydrate ingestion, during prolonged moderate-intensity exercise, there is a decline in liver glycogen content due to the stimulation of glycogenolysis to maintain delivery rates of endogenous blood glucose into the systemic circulation. When large amounts of glucose are ingested during exercise, exogenous glucose appearance rates displace almost all hepatic glycogenolysis as the source of systemic glucose appearance, leading to an attenuation or prevention of liver glycogen depletion. When large amounts of glucose plus fructose are ingested during exercise, there is, as yet no evidence that this leads to net liver glycogen storage, but rather the additional carbohydrate that is available is released into the systemic circulation for oxidation. Data pooled from maximal rates observed [52, 76–81].

**Fig. 2:**
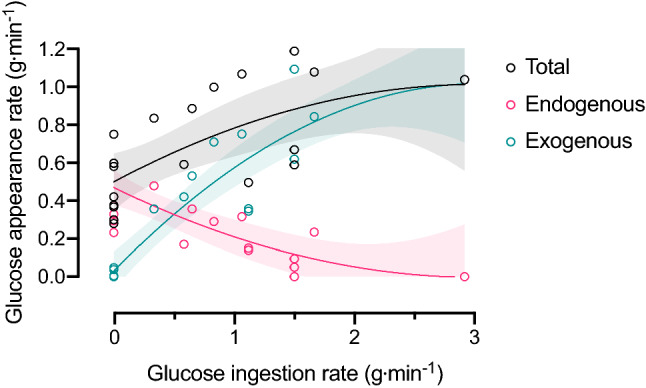
With increasing rates of glucose ingestion during prolonged moderate-intensity exercise, there is a progressive suppression of endogenous glucose appearance rates, which reach negligible rates when glucose is ingested at rates exceeding ~ 1.5 g⋅min^−1^. This is more than offset by an increase in exogenous glucose appearance rates, and therefore, total glucose appearance rates also increase with glucose ingestion rates to a maximum rate of 1.2 g⋅min^−1^

Once in the liver, the ingested CHO can have multiple fates, from oxidation or storage as glycogen, to conversion and release into the systemic circulation as alternative CHO (e.g., from fructose to glucose and/or lactate). During exercise without CHO feeding, the liver produces glucose via glycogenolysis and gluconeogenesis to maintain plasma glucose concentrations [56]. During prolonged (120 min), moderate-intensity exercise in the fasted state; the liver produces glucose at a rate of ~ 0.5 g⋅min^−1^ (30 g⋅h^−1^; Fig. 1), resulting in substantial liver glycogen depletion during exercise [47, 56]. Glucose ingestion during exercise suppresses hepatic glucose production in a dose-dependent manner. When glucose is ingested at rates above ~ 1.5 g⋅min^−1^ (90 g⋅h^−1^), hepatic glucose production can be completely abolished [57]. During this scenario, total systemic glucose appearance (and exogenous carbohydrate oxidation rates) appears to plateau at 1.0–1.2 g⋅min^−1^ (~ 72 g⋅h^−1^; Fig. 2), suggesting that all of the ingested glucose that is absorbed across the intestine is oxidized. Consistent with this, the ingestion of glucose at ~ 1.7 g⋅min^−1^ (~ 102 g⋅h^−1^) during prolonged (180 min) exercise results in no net changes in liver glycogen concentrations [47]. Since glucose–fructose mixtures can circumvent the saturation of SGLT1 to increase exogenous CHO availability from ~ 1.2 up to ~ 1.7 g⋅min^−1^ (42%). it has been speculated that the extra CHO available from fructose could lead to net hepatic glycogen storage during exercise [58]. Indeed, indirect evidence suggests that some fructose is directed to glycogen synthesis during exercise [59]. When adding 0.8 g⋅min^−1^ of fructose to 1.2 g⋅min^−1^ of glucose ingestion, ~ 0.48 g⋅min^−1^ of fructose may appear into the systemic circulation in the form of glucose and lactate (Fig. 1) [59], increasing systemic CHO availability. The remaining 0.32 g⋅min^−1^ is thought to undergo non-oxidative fructose disposal (i.e., glycogen storage) [59]. However, direct assessments of liver glycogen concentrations demonstrate no net increase in liver glycogen contents following the ingestion of glucose–fructose mixtures at ~ 1.7 g⋅min^−1^ during prolonged (180 min) endurance exercise [47]. This raises questions about the turnover of hepatic glycogen stores. It is possible that fructose is indeed directed to liver glycogen, but the metabolic and hormonal milieu of prolonged exercise may prevent net hepatic glycogen storage [56].

## Tertiary

The tertiary phase can be defined as the effects that CHO exerts when it becomes available in the systemic circulation. Maintaining CHO availability (and thereby preventing hypoglycaemia, defined as blood glucose concentrations  < 3.5 mmol/L) during exercise is important for both central (brain) as well as peripheral (muscle) function in maintaining carbohydrate oxidation to sustain exercise performance [60].

### Central Function

Upon entering the systemic circulation, glucose and lactate can be transported to and taken up by the brain. Glucose is (primarily) taken up through glucose transporters GLUT1 (*SLC2A1*) and GLUT3 (*SLC2A3*) and lactate via monocarboxylate transporters (MCTs) [61, 62]. At rest, glucose is the preferred energy substrate for the brain; therefore, a continuous supply of glucose from the systemic circulation is essential, as brain glycogen storage is limited [63, 64]. Resting plasma lactate concentrations of ~ 1.0 mmol⋅L^−1^ can contribute up to ~ 10% to brain metabolism, which can be further increased with higher plasma lactate concentrations (up to ~ 60% under supraphysiological plasma lactate concentrations) [65]. During exercise, when cerebral blood flow is increased, and lactate concentrations are raised, lactate may partially replace glucose as a substrate for energy provision [66–68]. The maintenance of sufficient glucose and/or lactate availability in the circulation and its subsequent oxidation appears to be essential to maintain normal brain function during exercise. Support for this thesis was provided by findings that exercise-induced hypoglycaemia causes central fatigue, which was counteracted by maintaining systemic glucose availability [69]. However, it is important to note that so far only limited data are available on the effects that glucose (and/or lactate) has on reducing central fatigue during exercise and, therefore, more research is warranted in this area [70]. From a more practical perspective, several studies have shown that ingesting CHO at ~ 30–60 g⋅min^−1^ during exercise can be important for factors such as decision-making and skill execution which are directly related to central function and represent key factors for optimal (intermittent) exercise performance [71–73].

### Peripheral Function

Glucose is transported to and taken up by the active muscle via GLUT1 (*SLC2A1*) and (primarily) GLUT4 (*SLC2A4*) through facilitated diffusion. GLUT4 is translocated from an intracellular microsomal GLUT4 pool to the cell membrane following (CHO-induced) insulin release and/or muscle contraction, thereby enabling a rapid increase in plasma glucose uptake within skeletal muscle tissue [74].

**Fig. 3:**
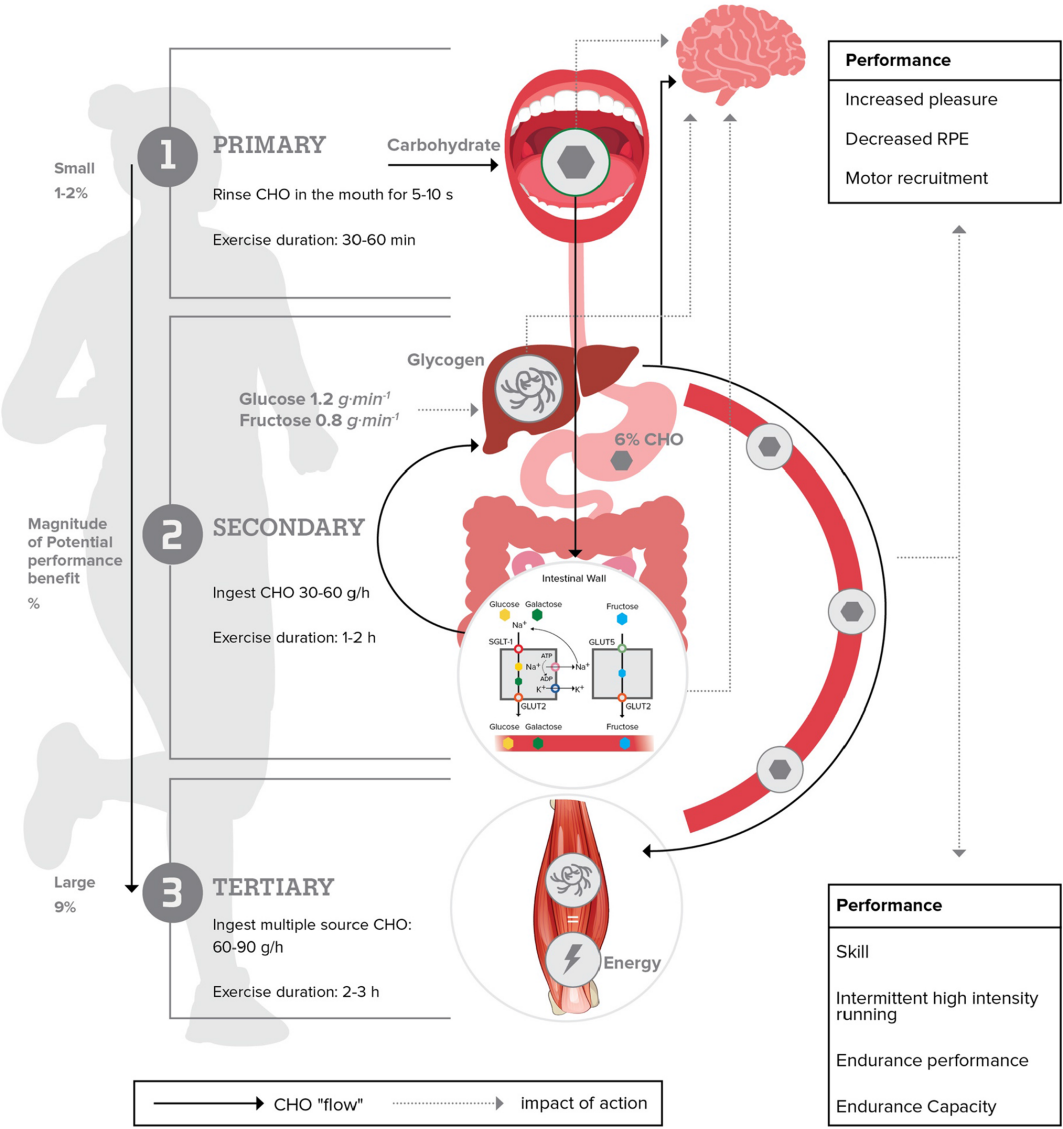
Schematic to capture the proposed primary (1), secondary (2), and tertiary (3) effects of CHO (grey hexagon) ingestion during exercise. Solid arrow indicates the movement of CHO in the body. Dotted arrow indicates the proposed mechanism of action. Carbohydrate intake guidelines are specific to performance-related goals over the associated duration and at exercise intensities > 70% *V*O_2_max

Whereas lactate appears to be predominantly oxidized within the active muscle [59, 75], glucose (after being phosphorylated by hexokinase into glucose-6-phosphate) has at least two distinct metabolic fates during exercise. Glucose within contracting muscle can be directly oxidized via a series of enzymatic reactions (i.e., glycolysis, TCA cycle, and oxidative phosphorylation) to generate adenosine triphosphate (ATP). It has been well established that when (only) glucose is ingested during exercise, that exogenous glucose can be oxidized at a maximal rate of ~ 1.0–1.2 g⋅min^−1^. However, these rates can be increased further to up to ~ 1.75 g⋅min^−1^ when a mixture of glucose (polymers) and fructose is ingested [44, 48, 76]. Therefore, the additional available (fructose-derived) glucose and lactate in the systemic circulation appear to be oxidized to generate ATP during exercise and maintain exercise performance [60]. Indeed, when performance is the goal, ingesting beverages with 0.5–1.0:1.0 fructose: glucose/maltodextrin ratio at a rate of ~ 90 g⋅h^−1^ during prolonged exercise (> 2.5–3.0 h) may improve endurance performance by ~ 8–9% [46, 77–79] (Fig. 3). It is important to note that high ingestion rates of CHO increase the risk of gastrointestinal discomfort, which can negatively impact performance. Therefore, athletes are encouraged to work towards guidelines and to practice CHO strategies in training [80].

The other potential metabolic fate of exogenous glucose is glycogen synthesis during exercise. Kuipers et al. (1987) provided evidence for glycogen resynthesis in non-active fast-twitch muscle fibers of trained cyclists who completed 3 h of low-intensity exercise (40% Wmax) during which they ingested ~ 2 L of a 25% maltodextrin drink (~ 167 g·h^−1^). Limited as this evidence is, it could contribute to the discussion on whether ‘glycogen sparing’ does or does not occur during prolonged exercise when participants ingest large amounts of CHO [81, 82]. Nonetheless, in a recent study, we did not find any muscle glycogen sparing in trained cyclists, who ingesting either glucose or sucrose (disaccharide consisting of glucose and fructose) during 3 h of cycling exercise at 50% Wmax [47]. There is no obvious explanation for the apparent discrepancy between studies, but it has been suggested that muscle glycogen sparing may occur in a time-dependent and/or fiber-type dependent manner [82]. Therefore, on balance, current evidence suggests that CHO ingestion during exercise primarily prevents liver, rather than muscle, glycogen depletion.

## Conclusion

It is widely acknowledged that ingested CHO can improve endurance capacity during prolonged exercise of moderate-to-high intensity by providing ample substrate for energy metabolism in skeletal muscle tissue. However, CHO is also the main substrate for energy metabolism in the brain and central nervous system (Fig. 3). Ingested CHO is first detected by receptors in the oral cavity and on the tongue. Its presence is relayed to reward and other centers in the brain resulting in a series of actions that include the release of insulin and enhanced endurance performance. After digestion, glucose is transported across the intestine into the systemic circulation in association with the active transport of Na^+^. The uptake of glucose in the GI tract seems to be limited by its intestinal uptake via SGLT1. As fructose is transported over the intestinal membrane via a different transporter protein, it has been reported that the combined ingestion of glucose and fructose can further increase the capacity to absorb exogenous CHO. Consequently, combining the ingestion of fructose and glucose can augment intestinal CHO uptake, increase post-prandial glucose availability, and increase exogenous carbohydrate oxidation rates by 40–50%. The hepatic glucose output resulting from the ingested CHO is distributed to both the brain and skeletal muscles so as to prevent hypoglycaemia and improve endurance performance capacity.
