# Correction to: Primary, Secondary, and Tertiary Effects of Carbohydrate Ingestion During Exercise

**DOI:** 10.1007/s40279-021-01489-8

**Published:** 2021-05-27

**Authors:** Ian Rollo, Javier T. Gonzalez, Cas J. Fuchs, Luc J. C. van Loon, Clyde Williams

**Affiliations:** 1 Gatorade Sports Science Institute, PepsiCo Life Sciences, Global R&D, Leicestershire, UK; 2 School of Sports Exercise and Health Sciences, Loughborough University, Loughborough, UK; 3 Department for Health, University of Bath, Bath, UK; 4 Department of Human Biology, NUTRIM School of Nutrition and Translational Research in Metabolism, Maastricht University Medical Centre+, Maastricht, The Netherlands

## Correction to: Sports Medicine (2020) 50(11):1863–1871 10.1007/s40279-020-01343-3

The Primary, Secondary, and Tertiary Effects of Carbohydrate Ingestion During Exercise, written by Ian Rollo, Javier T. Gonzalez, Cas J. Fuchs, Luc J. C. van Loon and Clyde Williams, was originally published electronically on the publisher’s internet portal on 16 September 2020 without open access. After publication in volume 50, issue 11, pages 1863–1871. With the author(s)’ decision to opt for Open Choice the copyright of the article changed on 27 May 2021 to © The Author(s) and the article is forthwith distributed under a Creative Commons Attribution 4.0 International License, which permits use, sharing, adaptation, distribution and reproduction in any medium or format, as long as you give appropriate credit to the original author(s) and the source, provide a link to the Creative Commons licence, and indicate if changes were made. The images or other third party material in this article are included in the article’s Creative Commons licence, unless indicated otherwise in a credit line to the material. If material is not included in the article’s Creative Commons licence and your intended use is not permitted by statutory regulation or exceeds the permitted use, you will need to obtain permission directly from the copyright holder. To view a copy of this licence, visit http://creativecommons.org/licenses/by/4.0/.

The original article has been corrected.

